# Leveraging Mann–Whitney U test on large-scale genetic variation data for analysing malaria genetic markers

**DOI:** 10.1186/s12936-022-04104-x

**Published:** 2022-03-09

**Authors:** Kah Yee Tai, Jasbir Dhaliwal, Vinod Balasubramaniam

**Affiliations:** 1 School of Information Technology, Monash University Malaysia, Subang Jaya, Selangor Malaysia; 2 Jeffrey Cheah School of Medicine & Health Sciences, Monash University Malaysia, Subang Jaya, Selangor Malaysia

**Keywords:** Malaria, Single nucleotide polymorphisms, Mann–Whitney U test, Descriptive statistics, Genetic markers

## Abstract

**Background:**

The malaria risk analysis of multiple populations is crucial and of great importance whilst compressing limitations. However, the exponential growth in diversity and accumulation of genetic variation data obtained from malaria-infected patients through Genome-Wide Association Studies opens up unprecedented opportunities to explore the significant differences between genetic markers (risk factors), particularly in the resistance or susceptibility of populations to malaria risk. Thus, this study proposes using statistical tests to analyse large-scale genetic variation data, comprising 20,854 samples from 11 populations within three continents: Africa, Oceania, and Asia.

**Methods:**

Even though statistical tests have been utilized to conduct case–control studies since the 1950s to link risk factors to a particular disease, several challenges faced, including the choice of data (ordinal vs. non-ordinal) and test (parametric vs. non-parametric). This study overcomes these challenges by adopting the Mann–Whitney U test to analyse large-scale genetic variation data; to explore the statistical significance of markers between populations; and to further identify the highly differentiated markers.

**Results:**

The findings of this study revealed a significant difference in the genetic markers between populations (p < 0.01) in all the case groups and most control groups. However, for the highly differentiated genetic markers, a significant difference (p < 0.01) was present for most genetic markers with varying p-values between the populations in the case and control groups. Moreover, several genetic markers were observed to have very significant differences (p < 0.001) across all populations, while others exist between certain specific populations. Also, several genetic markers have no significant differences between populations.

**Conclusions:**

These findings further support that the genetic markers contribute differently between populations towards malaria resistance or susceptibility, thus showing differences in the likelihood of malaria infection. In addition, this study demonstrated the robustness of the Mann–Whitney U test in analysing genetic markers in large-scale genetic variation data, thereby indicating an alternative method to explore genetic markers in other complex diseases. The findings hold great promise for genetic markers analysis, and the pipeline emphasized in this study can fully be reproduced to analyse new data.

**Supplementary Information:**

The online version contains supplementary material available at 10.1186/s12936-022-04104-x.

## Background

Malaria is a life-threatening disease caused by a parasite transmitted to humans by an infected female *Anopheles* mosquito bite. However, as the parasites involved are highly adaptable to nature, it is tremendously challenging to control the outbreak of this disease [1]. Moreover, risk prediction of this disease has proven challenging due to the combined effects of environmental and genetic factors. Thus, biological modelling research using genetic information for disease risk assessment has been supplemented by various approaches, including Genome-Wide Association Studies (GWAS).

GWAS is a popular approach that investigates associations between genetic information, in particular, specific Single Nucleotide Polymorphisms (SNPs), and disease. An SNP is the most common type of genetic variation of a disease and is henceforth a resistance or susceptibility marker. For example, a resistance marker can prevent the risk of developing the disease as well as reducing the severity of the symptoms. In contrast, a susceptibility marker increases the risk of developing the disease instead. Thus, SNPs can be used as genetic markers to represent disease-associated risk factors.

In malaria research, GWAS has successfully been applied in multiple malaria-endemic areas [2–11], where SNPs related to malaria resistance or susceptibility have been identified. The exponential growth in diversity and accumulation of SNP genotypes obtained from malaria-infected patients through GWAS such as Malaria Genomic Epidemiology Network (MalariaGEN) provides large-scale genetic variation data to explore the significant differences between genetic markers (risk factors) among populations. Thus, this study proposes using statistical tests to analyse the MalariaGEN data which comprising 20,854 samples from 11 populations within three continents: Africa, Oceania, and Asia.

A statistical test is a powerful tool widely used throughout the scientific research process to conclude from mass data, where it can be applied to study the relationship between risk factors and diseases [12]. For example, a statistical test can be used to explore the effect of exposure to risk factors between disease-infected patients (case) and healthy individuals (control). Note that statistical tests have been utilized to conduct case–control studies since the 1950s to link cigarette smoke to lung cancer [13]; and later to complex diseases such as breast cancer [14, 15], ischemic heart disease [16], type 2 diabetes [17] and asthma [18]. Thus, as risk factors play an important role in disease prediction and prevention, a statistical test can measure the statistical significance of the risk factors leading to diseases, and is the focus of the work here.

However, several challenges were faced when applying statistical tests to the work. The first major challenge is that researchers to date have studied the statistical significance of disease risk factors by applying the tests on ordinal clinical data, i.e., continuous variables and not genetic markers. This data includes demographic characteristics, lifestyle habits, physical measurements, medical records, family history of the related disease, and disease-related knowledge data. To overcome this challenge of non-ordinal data, the genetic risk scores were calculated from the genetic markers, i.e., SNPs. As these scores indicate the impact of genetic variations in populations, the genetic markers that contribute to malaria resistance or susceptibility between populations can be explored by adopting a statistical test. However, malaria is a complex disease involving various genetic markers from many different genes, which leads to the genetic basis of malaria resistance or susceptibility being complicated at multiple levels [19]. Therefore, this information was taken into consideration, and a statistical test was adopted to evaluate population associations with single locus genetic markers and multilocus genetic markers (by summing the genetic risk scores).

Choosing the correct statistical test is another challenge. There are two types of statistical tests: parametric test and non-parametric test. There has always been a dispute over the preferred test in medical research [20–22]. The main issue with parametric tests is that the results may be misleading if the normal distribution assumption is not met, leading to an erroneous conclusion [23]. Note that a parametric test can be applied to non-normally distributed data based on the central limit theorem. However, according to several studies [24–26], normally distributed data is an exception and not a rule in medical research. This is because real-world data usually follow a non-normal distribution [24], and by definition, ordinal data does not follow a normal distribution, which is also quite common in biomedical research [27]. A more than a decade-long study emphasized this point by analysing 630 studies from biomedical journals, and among them, non-parametric tests are more commonly applied in human studies [25]. Thus, descriptive statistics was first conducted to understand the characteristics of MalariaGEN data to obtain meaningful statistics in order to explore the genetic markers associated with malaria.

In malaria research, statistical tests have mainly been utilized to capture an individual's genes characteristics towards malaria resistance or susceptibility [28, 29]; and assess the consistency of expression profiles of genes between case and control [30]. To date, there is no research that uses statistical tests to analyse large-scale genetic variation data to explore significant differences of malaria genetic markers, particularly in the resistance or susceptibility of populations to malaria risk. Thus, it raises several research questions, including: (1) Are there significant differences in the likelihood of getting malaria between populations?; and (2) What genetic markers can be used to distinguish the population affected by malaria?. To answer these questions, the contributions of this paper are summarized as follows:

- Introduces how a statistical test can potentially be adopted to analyse genetic risk scores obtained from large-scale genetic variation data (non-ordinal data), i.e., SNPs genetic marker;
- Analyses statistical significance of malaria genetic markers between populations, and;
- Identifies highly differentiated genetic markers among populations.

## Methods

### Dataset and study population

The human GWAS data utilized in this study was generated from the MalariaGEN Consortial Project 1, entitled: “Genome-wide study of resistance to severe malaria in eleven populations”. The study comprised genotype data of 20,854 individuals from 11 worldwide populations: 10,791 severe malaria-affected individuals and 10,063 control subjects. Table 1 details the sample size of each population. The structure of the consortial project has been described in [31], and the collaboration of each partner’s studies and field sites was acknowledged on the MalariaGEN website http://www.malariagen.net/.

**Table 1:** Analysed populations and samples

Population	Case	Control	Sample size
Burkina Faso	807	639	1446
Cameroon	693	778	1471
Gambia	2807	2786	5593
Ghana	422	342	764
Kenya	1944	1738	3682
Malawi	1590	1498	3088
Mali	475	394	869
Nigeria	288	131	419
Tanzania	485	494	979
Vietnam	860	868	1728
Papua New Guinea	420	395	815
Total			20,854

Sample size indicates the total number of individuals for each population

### Candidate single nucleotide polymorphism

Through the review and analysis of 31 academic articles related to malaria research [3–11, 29, 32–52], a total of 122 SNPs were identified to be associated with malaria. However, of the 122 SNPs, 18 SNPs were excluded due to unreported effect size and unavailability in certain populations.

### Data preprocessing

Thus, 104 SNPs were extracted from the study subjects to analyse their genetic markers (Additional file 1). All unparseable values in the data, i.e., data type and standard format errors, are converted to null representations.

The Single Nucleotide Polymorphism database, in collaboration with EMBL-EBI European Variation Archive, assigns a unique ID to human genetic variation data, including SNPs [53]. These IDs are called rsIDs and appear in the format rs##. On the other hand, kgpIDs are identifiers created by Illumina during sequencing. There were 32 kgpIDs mapped to rsIDs, and 37 samples without severe malaria subtypes information were also removed. The subtype indicates the severity of malaria, which further influences the treatment plan.

The existing genotype imputation software, such as IMPUTE2 [54] and Beagle [55], usually impute missing genotypes based on publicly available reference datasets, such as 1000 Genomes Project or HapMap 3. However, in this case, imputation needs to be more specific, i.e., based on population group and severe malaria subtypes, as this study analyses the malaria risk of several populations.

Thus, a python program was developed to impute any missing genotypes based on the population group and severe malaria subtypes from the human GWAS data used in this study. In order to do so, the program first groups individuals based on their countries and then by their severe malaria subtypes. After that, the program compares a total of six SNPs for each missing genotype, i.e., three SNPs before and after the missing loci, and then imputes the missing genotype with the most common genotype data.

The dataset contains the genotype data of 104 SNPs formed by two alleles of *A* and *a*, usually expressed as *AA*, *Aa*, and *aa*. The genetic risk score for each genotype data was calculated to analyse the association between population and genetic markers and is described in the following section.

### Genetic risk score

The genetic risk score refers to a number reflecting the severity of the risk caused by specific genetic markers. In this study, the genetic risk score was calculated based on the genotype profile of each individual. This profile represents the impact of genetic variation on individuals in each population.

The most common approach to calculating genetic risk scores is weighted genetic risk scores (wGRS). The wGRS is calculated by multiplying the number of risk alleles (0, 1, 2) by the estimated effect size reported for each variant [56]. The logistic regression association tests method is used to estimate the variant effect size, described in the association test summary statistics available on the MalariaGEN website. However, this approach only considers the risk alleles and the effect size of the variant, which is not sufficient for malaria risk analysis in these aspects.

Some observations in the literature [57, 58] indicate that genotype patterns contribute to disease association, and extensive evidences have proven that sickle cell anemia traits can partially prevent malaria [19, 59–61]. The trait of sickle cell anaemia is caused by the recessive alleles in the haemoglobin gene. This means that an individual needs to have two copies of the recessive alleles—one from the mother and one from the father—to have this condition.

An individual tends to be resistant to the development of malaria if the two alleles are not identical (heterozygous). Conversely, an individual tends to be susceptible to the development of malaria if the two alleles are identical (homozygous). Therefore, the inclusion of genotype patterns is essential for differentiating genetic markers. Inspired by its importance, this study will include genotype frequency with wGRS to formulate more comprehensive genetic risk scores, namely wGRS + GF.

Genotype frequency indicates the relative frequency of a particular genotype in a population. The genotype frequency of each population is calculated from the genotype data by using the Hardy–Weinberg equation, as this equation calculates an individual’s genetic variation at equilibrium. The wGRS + GF is calculated by multiplying the genotype frequency by the wGRS mentioned above.

### Statistical analysis

#### Descriptive statistics

**Fig. 1:**
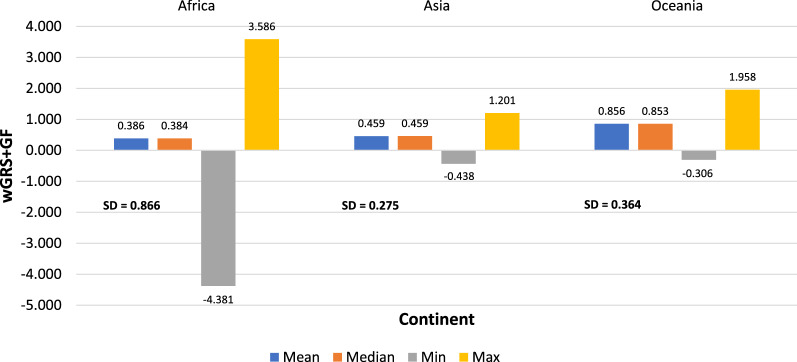
Descriptive statistics summary of genetic risk scores (wGRS + GF) based on continents, min = minimum, max = maximum, sd = standard deviation

**Fig. 2:**
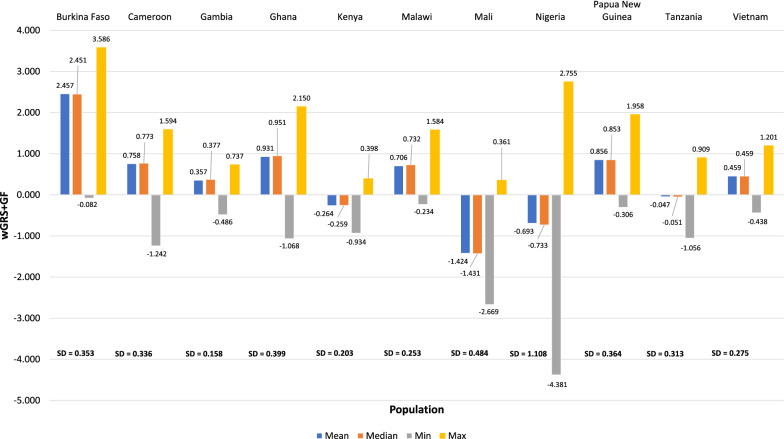
Descriptive statistics summary of genetic risk scores (wGRS + GF) based on populations, min = minimum, max = maximum, sd = standard deviation

**Fig. 3:**
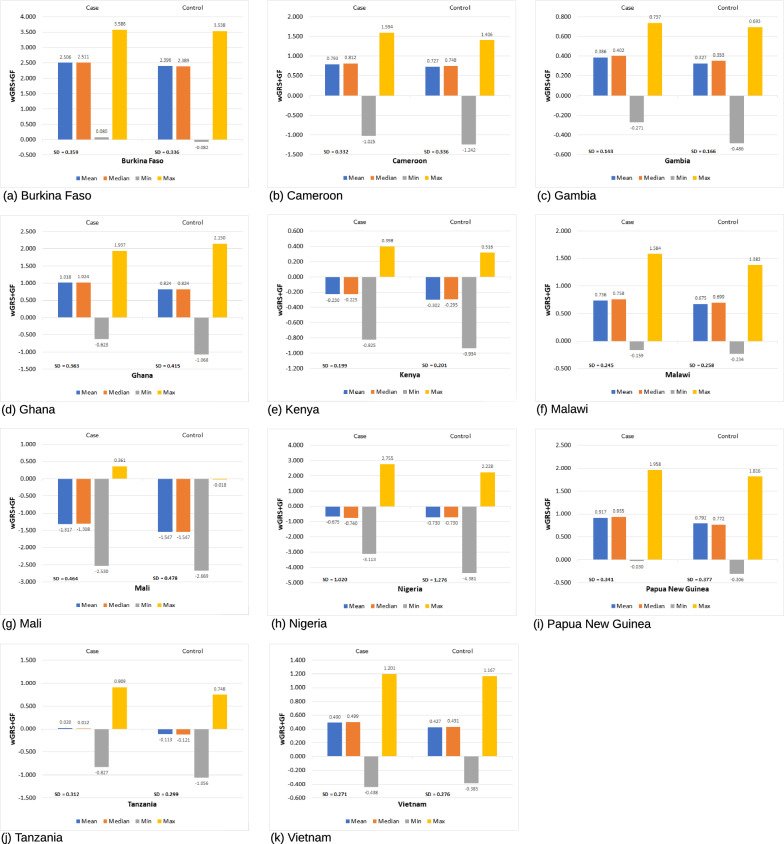
Descriptive statistics summary of genetic risk scores (wGRS + GF) based on case/control, min = minimum, max = maximum, sd = standard deviation

Descriptive statistics was performed to understand the characteristics of the data. The summary of mean, median, minimum, maximum, and standard deviation values of genetic risk scores based on continents, populations, and case/control is presented in Figs. 1, 2, and 3, respectively.

Based on the results in Fig. 1, the mean and median values are almost similar within continents, indicating that symmetric distributions exist. However, in Fig. 2, marginal differences were noticed between the population-based mean and median values. This further confirms the assumption of the population data being symmetrical and, therefore, the case/control distribution was explored within each population. As expected, the case/control distributions appear to be symmetrical for each population, as shown in Fig. 3. The Kurtosis and Skewness obtained are within the range of [− 0.2, + 10.1] and [− 2.4, + 0.3], and is the accepted range for symmetrical distribution where the absolute value of Kurtosis and Skewness should not be greater than 3 and 10 [62]. However, the sample sizes impact the Kurtosis and Skewness values, and in this case, a large-scale genetic variation data with different characteristics was used. Therefore, based on the results obtained above, parametric tests such as Welch’s t-test and non-parametric tests such as the Mann–Whitney U test was further explored on the case/control data. Initial exploration results indicated no significant differences in the p-values obtained from both the tests via one-way Analysis of Variance (ANOVA) test with p < 0.05.

Mann–Whitney U test is based on the median, whereas Welch’s t-test is based on the mean. However, median is the preferred measurement when data is measured on an ordinal scale, which is most suitable for real-world data [63]. Normally distributed data in medical research is an exception because real-world data is usually non-normally distributed and contains ordinal data [27]. Therefore, Mann–Whitney U test was adopted for the work here.

#### Mann–Whitney U test

The Mann–Whitney U test was implemented in Python using the pingouin.mwu() function [64] to test the null hypothesis of this study, i.e., there will be no statistically significant differences in genetic risk scores by population groups. This function takes two data samples as parameters and uses the median as a measure of central tendency, and then returns the test results with a p-value to indicate the statistical significance. All analyses utilized a significance level of p < 0.01 because it is a commonly used p-value for studying statistical significance in biomedical research [65]. The p = 0.00E+00 is considered as very significant differences (p < 0.001).

The statistical analysis for the work here comprises two parts. Part 1 involves general analysis to evaluate the association between the population and the cumulative effects of SNPs to study the statistical significance of multilocus genetic markers among populations. The cumulative effect is calculated by summing the genetic risk scores of all the 104 SNPs. On the other hand, Part 2 involves a detailed analysis to evaluate the association between the population and the genetic risk score of each SNP (single locus) to identify the highly differentiated genetic markers between populations. In other words, Part 2 analyses the effect of each SNP instead of the combined effect of all the 104 SNPs. Both parts are analysed based on two groups: case and control, and are performed based on wGRS + GF as the genetic risk score described in the previous section.

**Fig. 4:**
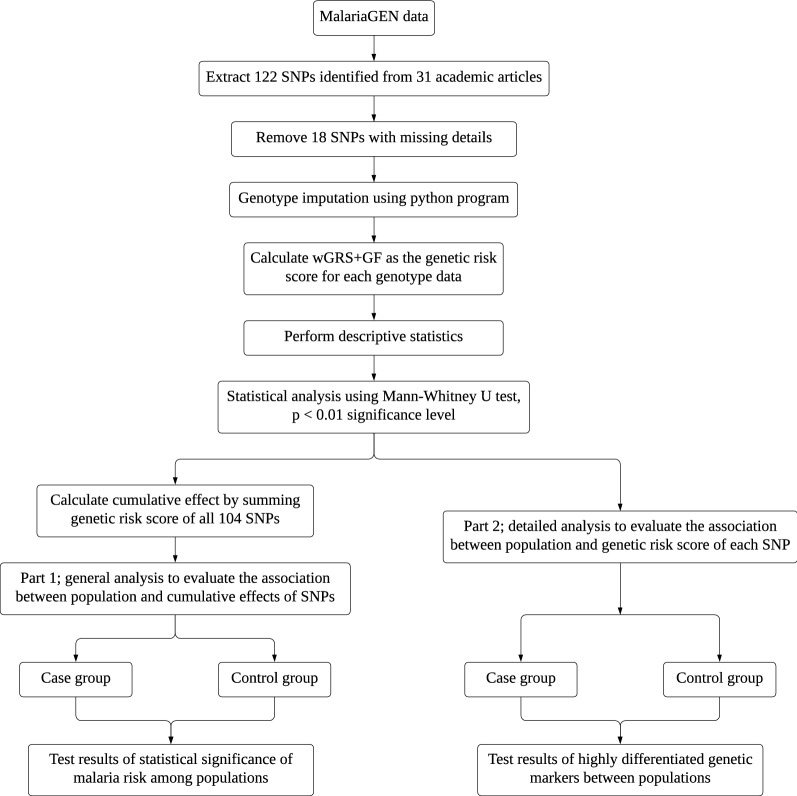
Methodology pipeline for statistical analysis of malaria genetic markers

Figure 4 shows the methodology pipeline in detail. All code was developed using the Python programming language, and simulations were performed on a machine with a 2.9 GHz Dual-Core Intel Core i5 processor and 8 GB of memory.

## Results

This section describes the experimental results based on Part 1 and Part 2.

### Part 1: general analysis

The first part is to study the statistical significance of multilocus genetic markers among populations by evaluating the association between the population and the cumulative effects of 104 SNPs.

#### Analysis of case group results

Table 2 shows the test results with p-values for the case group. A significant difference (p < 0.01) was present for all populations. Of particular note is the very significant differences (p < 0.001) between Burkina Faso and Gambia, Burkina Faso and Kenya, Burkina Faso and Malawi, Gambia and Kenya, Gambia and Malawi, Kenya and Malawi, Kenya and Vietnam.

**Table 2:** Population case groups test results with p-values

Country	Burkina Faso	Cameroon	Gambia	Ghana	Kenya	Malawi	Mali	Nigeria	Papua New Guinea	Tanzania	Vietnam
Burkina Faso		1.01E−242	0.00E+00	9.98E−181	0.00E+00	0.00E+00	6.11E−192	7.24E−133	2.14E−179	8.12E−199	4.74E−271
Cameroon	1.01E−242		2.97E−276	4.10E−27	4.15E−305	5.42E−10	4.46E−179	5.52E−85	1.28E−07	5.20E−159	2.64E−97
Gambia	0.00E+00	2.97E−276		9.24E−198	0.00E+00	0.00E+00	1.15E−256	1.94E−78	1.21E−176	4.67E−140	6.59E−33
Ghana	9.98E−181	4.10E−27	9.24E−198		3.29E−221	1.04E−58	1.18E−144	6.39E−82	1.22E−05	6.80E−136	6.21E−113
Kenya	0.00E+00	4.15E−305	0.00E+00	3.29E−221		0.00E+00	8.26E−227	1.09E−23	1.45E−224	3.59E−65	0.00E+00
Malawi	0.00E+00	5.42E−10	0.00E+00	1.04E−58	0.00E+00		8.48E−233	6.63E−99	1.46E−24	4.41E−207	3.52E−96
Mali	6.11E−192	4.46E−179	1.15E−256	1.18E−144	8.26E−227	8.48E−233		7.64E−21	7.39E−144	8.30E−149	3.24E−195
Nigeria	7.24E−133	5.52E−85	1.94E−78	6.39E−82	1.09E−23	6.63E−99	7.64E−21		1.15E−77	3.74E−31	1.68E−71
Papua New Guinea	2.14E−179	1.28E−07	1.21E−176	1.22E−05	1.45E−224	1.46E−24	7.39E−144	1.15E−77		6.01E−133	1.43E−84
Tanzania	8.12E−199	5.20E−159	4.67E−140	6.80E−136	3.59E−65	4.41E−207	8.30E−149	3.74E−31	6.01E−133		2.68E−113
Vietnam	4.74E−271	2.64E−97	6.59E−33	6.21E−113	0.00E+00	3.52E−96	3.24E−195	1.68E−71	1.43E−84	2.68E−113	

#### Analysis of control group results

On the other hand, Table 3 presents the test results with p-values for the control group. In contrast to Table 2 that had significant differences (p < 0.01) for all populations, no significant differences were found between the Cameroon and Papua New Guinea, Ghana and Papua New Guinea populations. Moreover, very significant differences (p < 0.001) were found between Burkina Faso and Gambia, Cameroon and Kenya, Gambia and Kenya, Gambia and Malawi, Kenya and Malawi, Kenya and Vietnam.

**Table 3:** Population control groups test results with p-values

Country	Burkina Faso	Cameroon	Gambia	Ghana	Kenya	Malawi	Mali	Nigeria	Papua New Guinea	Tanzania	Vietnam
Burkina Faso		2.46E−229	0.00E+00	7.85E−146	2.17E−306	3.09E−292	7.17E−161	2.26E−71	7.89E−160	4.33E−183	1.67E−240
Cameroon	2.46E−229		1.19E−300	2.42E−05	0.00E+00	4.35E−09	3.80E−171	1.29E−43	–	3.95E−173	1.13E−104
Gambia	0.00E+00	1.19E−300		8.19E−130	0.00E+00	0.00E+00	6.23E−227	3.43E−29	1.82E−130	3.53E−178	1.86E−24
Ghana	7.85E−146	2.42E−05	8.19E−130		1.02E−173	1.55E−15	1.03E−120	1.52E−38	–	2.86E−115	2.76E−64
Kenya	2.17E−306	0.00E+00	0.00E+00	1.02E−173		0.00E+00	3.40E−201	4.96E−05	5.40E−209	8.30E−42	0.00E+00
Malawi	3.09E−292	4.35E−09	0.00E+00	1.55E−15	0.00E+00		2.13E−205	1.01E−44	7.73E−08	6.14E−219	3.81E−91
Mali	7.17E−161	3.80E−171	6.23E−227	1.03E−120	3.40E−201	2.13E−205		6.97E−14	1.61E−130	2.13E−140	1.62E−178
Nigeria	2.26E−71	1.29E−43	3.43E−29	1.52E−38	4.96E−05	1.01E−44	6.97E−14		4.39E−39	1.97E−08	1.59E−30
Papua New Guinea	7.89E−160	–	1.82E−130	–	5.40E−209	7.73E−08	1.61E−130	4.39E−39		4.07E−130	7.77E−58
Tanzania	4.33E−183	3.95E−173	3.53E−178	2.86E−115	8.30E−42	6.14E−219	2.13E−140	1.97E−08	4.07E−130		1.77E−138
Vietnam	1.67E−240	1.13E−104	1.86E−24	2.76E−64	0.00E+00	3.81E−91	1.62E−178	1.59E−30	7.77E−58	1.77E−138	

### Part 2: detailed analysis

The second part is to identify the highly differentiated genetic markers between populations by evaluating the association between the population and the genetic risk score of each SNP (single locus).

#### Analysis of case group results

Additional file 2 shows highly differentiated genetic markers with very significant differences (p < 0.001) between populations, and the test results of each SNP for the case group are summarized in Additional file 3. Significant difference (p < 0.01) was present for most genetic markers with varying p-values between the populations.

#### Analysis of control group results

Following this, Additional file 4 shows highly differentiated genetic markers with very significant differences (p < 0.001) between populations, and the test results of each SNP for the control group are summarized in Additional file 5. Similar to the case group, a significant difference (p < 0.01) was present for most genetic markers with varying p-values between the populations.

## Discussion

Up-to-date, not many statistical analysis studies have been carried out to study the relationship between malaria risk and populations. However, these studies utilized environmental data such as low altitude, high temperature, and humidity with malaria incidences [66]. For example, humidity in a region can affect the survival rate of mosquitoes [67], and deforestation can significantly increase the spread of malaria [68, 69]. On the other hand, in regards to resistance or susceptibility to the risk of malaria, several risk factors have been identified, including genetic variation. Recall that an individual might prevent disease risk with a resistance marker; while increase disease risk with a susceptibility marker. Human genetics and epidemiological studies have confirmed that human genetic variation contributes differently to diseases due to differences in resistance or susceptibility levels [19, 70, 71].

Genetic markers are essential in providing a basis for understanding genetic differences between populations and malaria risk. These markers have been utilized to characterize the genetic composition and complexity of the disease. However, no study has analysed the significant differences of the genetic markers. The initial exploration results based on descriptive statistics indicated case/control distribution data to be symmetric. However, the Mann–Whitney U test was chosen over Welch’s t-test, as there are no significant differences in the p-values obtained from both the tests via one-way ANOVA test. Moreover, prior studies recommended the use of the median for real-world data.

The Mann–Whitney U test was performed to study the statistical significance of genetic risk scores by population groups. This study introduces a statistical test to analyse large-scale genetic variation data of case and control groups to study the statistical significance of genetic markers. In particular, the human GWAS datasets obtained from MalariaGEN were analysed, which contains 11 worldwide populations.

To formulate a more comprehensive genetic risk score, genotype frequency was combined with wGRS. This score represents the impact of genetic variation for each individual, which further contributes to the population genetic risk score. Inclusion of genotype frequency is essential because studies have shown that genotype patterns play a crucial role in malaria resistance or susceptibility. The performed statistical tests were based on the case and control groups with a significance level of p < 0.01.

The association between population and cumulative effects of all the 104 SNPs was evaluated to study the statistical significance of multilocus malaria genetic markers between populations. The test results revealed a significant difference (p < 0.01) for all populations in the case group. Likewise, in the control group, a significant difference (p < 0.01) was present for all populations, except between Cameroon and Papua New Guinea, Ghana and Papua New Guinea populations. These results further confirm that genetic markers vary between populations.

The significant differences in genetic variation used as markers to distinguish populations have not yet been discovered. Therefore, the association between the population and the genetic risk score of each SNP (single locus) was evaluated to identify the highly differentiated genetic markers. The test results showed a significant difference (p < 0.01) for most genetic markers between the case and control groups. Moreover, the results show that the p-value of the genetic markers vary between populations. More highly differentiated genetic markers with very significant differences (p < 0.001) were observed in the Gambia, Kenya, and Malawi populations. In addition, several genetic markers have very significant differences (p < 0.001) across all populations, while others were only observed between certain specific populations. Also, several genetic markers have no significant differences between populations. The findings indicate that the highly differentiated genetic markers that contribute to malaria risk differ between populations due to genetic differences.

This study has presented a method to analyse large-scale genetic variation data through the Mann–Whitney U test to explore genetic markers of malaria. Many previous studies have analysed malaria genetic markers, focusing on either resistance [6, 37, 59] or susceptibility markers [8, 32, 41]. However, no study combines the resistance and susceptibility markers and then analyse them together. For this study, it is important to combine these markers, as there is an interest in exploring the statistical significance of markers between populations, as well as identifying the highly differentiated markers.

Besides that, previous studies have used statistical tests to explore malaria risk depending on the purpose of analysis. For example, the Chi-square test was used to estimate the prevalence of specific genes in malaria-endemic populations [72, 73]; Student t-test was used to study the association between malaria susceptibility and genetic variation in the immune system [74]; Fisher’s exact test was used to analyse differences between clinical groups of children with acute malaria in categorical parameters [75]. However, these tests are not suitable for this study for the following reasons. The Chi-square test has limitations in interpreting large sample sizes [76], while the Student t-test requires normally distributed data [26]. Finally, Fisher’s exact test is best with small-size samples [77].

On the other hand, this study support prior research that indicates the Mann–Whitney U test is the preferred test for analysing real-world medical data [27], especially in this study’s case of ordinal data consisting of genetic risk scores. Moreover, the Mann–Whitney U test, which is based on median, is the preferred test as prior research [63] has also indicated that median is the preferred measurement for ordinal data. This is an important result as it establishes the Mann–Whitney U test as the most appropriate statistical test to be adopted for this study. It is believed that these findings hold great promise for genetic markers analysis and may serve as a robust tool for further studies analysing genetic markers based on ordinal data in other diseases. To further interpret the complexity of malaria, a future study that integrates large-scale environmental data and genetic variation data for statistical testing may be considered. Besides genetic markers, environmental factors also play an essential role in a region contracting malaria. Therefore, understanding the population genetic markers and environmental variables in a region will help further characterize the significant differences in malaria risk.

## Conclusions

This study conducted a malaria risk analysis based on the MalariaGEN human GWAS datasets that contain 11 populations. More precisely, a statistical test was adopted to explore genetic risk scores obtained from SNPs genetic markers. The analysis of the association between population and the cumulative effects of SNPs was carried out to study the statistical significance of multilocus malaria genetic markers. Then, the association between the population and the genetic risk score of each SNP (single locus) was further explored to identify the highly differentiated genetic markers. The findings indicate that populations have different genetic markers affecting malaria resistance or susceptibility levels. Therefore, there are significant differences in the likelihood of malaria infection among populations. It is believed that the findings of this study can help further characterize the complexity of the disease and provide additional knowledge regarding the association of malaria risk among populations. To a larger extent, the study has shown a promising method that demonstrates how statistical tests can be adopted to analyse large-scale genetic variation data to explore genetic markers associated with complex diseases.

## Supplementary Information

**Additional file 1. **General information on the 104 SNPs used in this study.

**Additional file 2. **Highly differentiated genetic markers with very significant differences (p < 0.001) analysed on population case groups.

**Additional file 3. **Population case groups test results of each SNP with their corresponding p-value.

**Additional file 4. **Highly differentiated genetic markers with very significant differences (p < 0.001) analysed on population control groups.

**Additional file 5. **Population control groups test results of each SNP with their corresponding p-value.

## Data Availability

The datasets analysed during the current study are available in the MalariaGEN Consortial Project 1 (https://www.malariagen.net/projects/consortial-project-1).

## Abbreviations

ANOVA: Analysis of variance
GWAS: Genome-Wide Association Studies
MalariaGEN: Malaria Genomic Epidemiology Network
SNP: Single nucleotide polymorphisms
wGRS: Weighted genetic risk scores
